# Eyeing the brain

**DOI:** 10.1007/s00401-016-1628-z

**Published:** 2016-10-15

**Authors:** M. Francesca Cordeiro

**Affiliations:** 1 Institute of Ophthalmology, UCL Institute of Ophthalmology, 11-43 Bath Street, London, EC1V 9EL UK; 2 ICORG, Imperial College London, London, UK; 3 Imperial College NHS Trust, Western Eye Hospital, London, UK

The involvement of the retina in CNS disease is increasingly recognized in the area of neurodegeneration, as there is accumulating evidence showing that similar mechanisms occur in the eye and the brain. These neuropathological processes ultimately lead to neuronal cell death and include ischaemia, inflammation, mitochondrial dysfunction (including oxidative stress), deposition of misfolded proteins, and changes in fluid dynamics [aqueous and cerebrospinal fluid (CSF)] [1, 5, 6, 9–11].

**Fig. 1:**
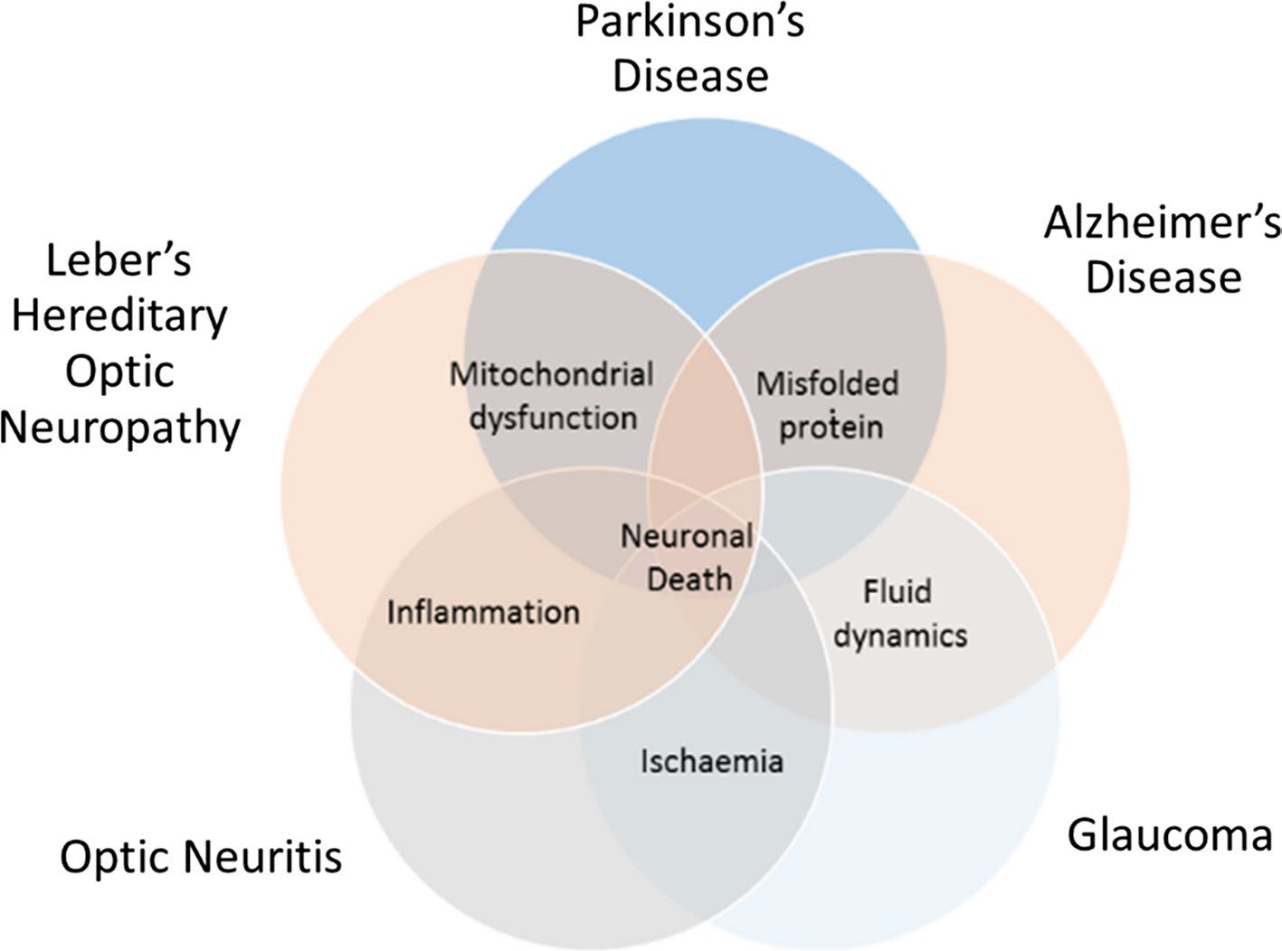
Examples of neurodegenerative eye and brain diseases illustrating overlap of key neuropathological mechanisms

Although common to some degree in all neurodegenerative conditions, specific events are associated more closely with certain diseases. Hence, deposition and aggregation of the misfolded proteins alpha-synuclein and beta-amyloid are believed to be key in their development in Parkinson’s disease (PD) and Alzheimer’s disease (AD), respectively [4]; mitochondrial dysfunction is particularly associated with LHON (Leber’s Hereditary optic neuropathy) and PD [12]; an important element of optic neuritis and LHON is inflammation [2, 14]; both optic neuritis and glaucoma include an ischemic aetiology [3, 8]; and finally, abnormal fluid dynamics is heavily implicated in glaucoma (through intraocular pressure) [3] and AD (CSF clearance) [7]. These overlaps are illustrated in the Venn diagram above (Fig. 1).

This issue of Acta Neuropathologica includes a cluster of three review papers on different aspects of neurodegeneration involving the retina, from experts in ophthalmology, neuro-ophthalmology, and neuroscience and covering glaucoma [3], Alzheimer’s disease [7], and LHON [13]. In each case, they address the neuropathological mechanisms that highlight why the retina may serve as a valuable model to study brain disease. They include molecular advances, evidence for commonalities between brain and eye changes, cell death processes, and recent updates on targets for therapy. Furthermore, they promote the idea that due to its accessibility, the eye can be a tool through which disease activity and treatment response can be assessed with widespread applications for neurological disease. This cluster will hopefully be of interest to neuropathologists, ophthalmic pathologists, neuroscientists, and neurologists intrigued by how and why it might be possible to eye the brain.
